# Genome Wide Association Mapping of Spot Blotch Resistance at Seedling and Adult Plant Stages in Barley

**DOI:** 10.3389/fpls.2020.00642

**Published:** 2020-05-25

**Authors:** Andrea Visioni, Sajid Rehman, Shyam Saran Viash, Shiw Pratap Singh, Ram Vishwakarma, Sanjaya Gyawali, Ayed M. Al-Abdallat, Ramesh Pal Singh Verma

**Affiliations:** ^1^Biodiversity and Crop Improvement Program, International Center for Agricultural Research in the Dry Areas, Rabat, Morocco; ^2^Department of Mycology and Plant Pathology, Institute of Agricultural Sciences, Banaras Hindu University, Varanasi, India; ^3^Department of Plant Pathology, Narendra Dev University of Agriculture and Technology, Faizabad, India; ^4^Vegetable Seed Pathology Department, Washington State University, Northwest Washington Research and Extension Center, Mount Vernon, WA, United States; ^5^Department of Horticulture and Crop Science, Faculty of Agriculture, The University of Jordan, Amman, Jordan

**Keywords:** spot blotch, AUDPC, barley, *Cochliobolus*, GWAM, resistance

## Abstract

Barley spot blotch (SB) caused by *Cochliobolus sativus* is one of the major constrains to barley production in warmer regions worldwide. The study was undertaken to identify and estimate effects of loci underlying quantitative resistance to SB at the seedling and adult plant stages. A panel of 261 high input (HI-AM) barley genotypes consisting of released cultivars, advanced breeding lines, and landraces, was screened for resistance to SB. The seedling resistance screening was conducted using two virulent isolates from Morocco (ICSB3 and SB54) while the adult plant stage resistance was evaluated at two hot spot locations, Faizabad and Varanasi, in India under artificial inoculation using a mixture of prevalent virulent isolates. The HI-AM panel was genotyped using DArT-Seq high-throughput genotyping platform. Genome wide association mapping (GWAM) was conducted using 13,182 PAV and 6,311 SNP markers, for seedling and adult plant resistance. Both GLM and MLM model were employed in TASSEL (v 5.0) using principal component analysis and Kinship Matrix as covariates. Final disease rating and Area Under Disease Progress Curve (AUDPC) were used for the evaluation of adult stage plant resistance. The GWAM analysis indicated 23 QTL at the seedling stage (14 for isolate ICSB3 and 9 for isolate SB54), while 15 QTL were detected at the adult plant stage resistance (6 at Faizabad and 9 at Varanasi) and 5 for AUDPC based resistance at Varanasi. Common QTL at seedling and adult plant stages were found across all barley chromosomes. Seedling stage QTL explained together 73.24% of the variance for seedling resistance to isolate ICSB3 and 49.26% for isolate SB54, whereas, QTL for adult plant stage resistance explained together 38.32%, 44.09% and 26.42% of the variance at Faizabad and Varanasi and AUDPC at Varanasi, respectively. Several QTL identified in this study were also reported in previous studies using bi-parental and association mapping populations, corroborating our results. The promising QTL detected at both stages, once validated, can be used for marker assisted selection (MAS) in SB resistance barley breeding program.

## Introduction

Spot blotch (SB) of barley (*Hordeum vulgare* L.), also commonly referred as leaf blight, and is caused by *Cochliobolus sativus* [anamorph: *Bipolaris sorokiniana* (Sacc.) Shoem.]. It is one of the major concerns in South Asia including China, Nepal, Pakistan, Bangladesh and the humid north eastern regions of India (Kumar et al., 2007; Chand et al., 2008; Singh et al., 2009; Vaish et al., 2011; Prasad et al., 2013). In addition, SB is also considered as a serious threat to barley production in the upper Midwest of the United States and the prairie provinces of Canada (Clark, 1979; Ghazvini and Tekauz, 2007). Recently SB has been identified in the warm regions of North Africa, especially in Morocco (Rehman et al., unpublished data). The yield losses of up to 36% in susceptible cultivars under disease conducive conditions have been reported in the United States with reduction in malting quality (Clark, 1979). In a disease survey of 2003–2006 in eastern Uttar Pradesh, Bihar states of India, SB was recovered from 63% of the blighted leaves. In addition, during field trials 42.5% SB severity was recorded on susceptible barley variety RD 2503 even after three fungicide treatments (Singh et al., 2009). Furthermore, Vaish et al. (2011) reported 21.3% SB incidence on barley in a survey conducted in the cold arid Trans-Himalayan region of India, where barley is grown in summer season from May to September. SB has not been reported before from Morocco, but recent disease surveys have shown its presence. The Moroccan SB isolates have shown a diversity of virulence on the set of 12-differential barley genotypes tested (Rehman et al., unpublished data). Therefore, understanding host-pathogen interaction at genetic level is quite important on identifying and deploying SB resistance. The aggressiveness of SB in South Asia and North Africa is a serious threat to barley cultivation in these regions including Morocco and India.

Although fungicide applications have been reported effective to control SB (Kiesling, 1985; Anonymous, 2011), but their use increases the cost of barley cultivation. Host resistance is considered important for Asian and African regions to control foliar blights where barley is grown by small holder farmers in marginal lands under low-input conditions. Thus, host resistance is widely considered to be the most sustainable and economical method for managing SB in barley (Wilcoxson et al., 1990). Remarkably stable SB resistance from NDB 112 (developed from a cross CIho 7117-77//Kindred by Wilcoxson et al. (1990) has protected six-row malting cultivars for the last 50 years in the Upper Midwest United States. Despite the transfer of all resistance loci into two-row barley like Bowman (PI483237), stable resistance like NDB 112 has not been observed and a differential expression of resistance loci in entirely different genetic background has been attributed to it (Fetch and Steffenson, 1994; Bilgic et al., 2005). The association mapping (AM) has advantages over bi-parental mapping like increased resolution for mapping QTL, greater diversity of alleles and being faster and efficient (Lander and Botstein, 1986; Buntjer et al., 2005; Yu and Buckler, 2006).

Several studies have identified QTL to SB resistance by using diverse wild and cultivated germplasm against SB pathotype 1, 2, and 7 (Roy et al., 2010; Zhou and Steffenson, 2013; Wang et al., 2017). Roy et al. (2010) has shown nicely the additive effect of each QTL on SRT and APR. Breeding lines carrying resistance allele of one QTL *Rcs-qtl-1H-11_10764* reduced infection rate (IR) from 0 to 20% and disease severity from 20 to 29%. Barley lines carrying two QTL *Rcs-qtl-1H-11_10764* and *Rcs-qtl-3H-11_10565* reduced IR from 5 to 31% and disease severity from 52 to 56%. Furthermore, barley lines carrying three *QTL Rcs-qtl-1H-11_10764, Rcs-qtl-3H-11_10565, Rcs-qtl-7H-11_20162* showed 47% lower IR and 83% lower disease severity when compared with lines lacking any of three QTL. Similar findings on additive effects of QTL for stripe rust of barley have been reported (Castro et al., 2003).

Mapping of effective SB resistance in South Asian and North African barley germplasm is still lagging behind (Gyawali et al., 2018) resulting in slow progress in employing marker-assisted selection of SB resistance to pyramid effective genes against other foliar pathogens of barley. The present study was taken up to map SB resistance in High Input Association Mapping (HI-AM) panel using genome wide association mapping (GWAM) approach at the seedling and adult plant stages.

## Materials and Methods

### Plant Materials

The HI-AM panel used in this study is composed of 261 spring barley genotypes (released cultivars from different countries; advanced breeding lines from ICARDA’s barley breeding program, and landraces from GenBank). The set is named as HI-AM (High Input Association Mapping) panel as most of barley genotypes were targeted toward optimum management (supplemental irrigation and fertilizer) conditions. Out of the 261 genotypes (172 two-row and 89 six-row types), 124 were from ICARDA’s barley breeding program (50 two-row and 74 six-row type), 32 from Europe (28 two-row and 4 six-row type), 34 from North America (28 two-row and 6 six-row type), 67 from South America (62 two-row and 5 six-row type), and 4 from Australia (all two-row type). The full list of genotypes is available in Supplementary Table S1.

### Screening for Seedling Resistance With Moroccan *C. sativus* Isolates

The seedling resistance test (SRT) for HI-AM panel was done with two *C. sativus* isolates under controlled conditions in the growth chamber at the International Center for Agricultural Research in the Dry Areas (ICARDA), Rabat, Morocco. These *C. sativus* isolates were collected from farmer’s field in Morocco during the disease survey of 2015 and were preserved as mono-conidial isolates in −80°C until further use (Supplementary Table S2). Two *C. sativus* isolates (ICSB3 and SB54) were classified into pathotypes by using three differential barley cultivars (NDB5883, Bowman, ND B112) as described by Fetch and Steffenson (1999). The isolate ICSB3 belongs to pathotypes 7 (virulent on NDB5883, Bowman, and ND B112) and SB54 belongs to pathotype 3 (virulent on ND B5883, and Bowman) (Rehman et al., unpublished data). To produce inoculum, lyophilized agar plugs of mono-conidial isolates were incubated on V8PDA (Vegetable juice 200 ml, potato dextrose agar 10 g, bacteriological agar 10 g) in the dark for 4–5 days at 20°C followed by incubation at 20°C with 12 h light/12 h dark photoperiod for 7–8 days. Further, the V8PDA plates were flooded with 5–10 ml of sterile distilled water and the conidia were harvested by rubbing the agar surface with sterile specula followed by filtration with double layer of cheese cloth. The spore density was adjusted to 5000 conidia ml^–1^ supplemented with the surfactant (0.01% of Tween 20).

About 4–5 seeds of each barley genotype were sown in peat moss in a single cone of 3.8 cm diameter and 14 cm depth (Stuewe & Sons, Inc., OR, United States) supplemented with 14–14–14 NPK and the seedlings were raised in the growth chamber with photoperiod of 16 h light/8 h dark at 20°C. Each tray containing 96 test genotypes along with resistant (ND B112) and susceptible checks (Annoucer [a Moroccan variety highly susceptible to SB]), was inoculated with 100 ml of spore suspension with hand held sprayers (0.2 ml/seedling) till run off followed by incubation under 100% relative humidity for 24 h in the dark at 20°C. After 24 h, the seedlings were transferred to growth chamber under same conditions as described earlier (Fetch and Steffenson, 1999). The experiment was laid out for three replications using a randomized complete block design.

A disease rating scale of 0–9 (Fetch and Steffenson, 1999), was used to evaluate the level of disease resistance at 10 days post inoculation (dpi). Based on the infection responses barley genotypes were grouped as immune (0), resistant (1–3), moderately resistant (4–5), moderately susceptible (6), susceptible (7–8) or highly susceptible (9) as described by Fetch and Steffenson (1999). Two independent replications of HI-AM were inoculated with each SB pathotype and the mean infection types of two replications was used in further analysis.

### Screening for Spot Blotch Resistance at the Adult Stage

Resistance at adult plant stage was assessed in three trials, for 2 years, at two different locations. In 2013–2014 growing season, a set of 261 barley genotypes (HI-AM panel) including two standard checks, Rihane-03 and VMorales, was sown in first week of December 2013 at the Agricultural Research Farms of the Banaras Hindu University (BHU), 25.2677°N, 82.9913°E, Varanasi, and at Narendra Dev University of Agriculture and Technology (NDUAT), 26.7732°N, 82.1442°E, Faizabad, both in Uttar Pradesh, India. These genotypes were sown in a 1-m row using augmented block design with a highly susceptible genotype, “RD2503” repeated at interval of 20 test genotypes. RD2503 was selected as a SB susceptible check because it showed highly susceptible reactions (IR = 8–9 on 0–9 scale) at the seedling stage and 99 score (double-digit score) of SB severity at the adult stages in the field. Further, RD2503 was grown as long paired row perpendicular to the test plots as spreader rows on either side. The SB isolates (locally collected and maintained as mono-conidial pure culture at BHU and NDUAT) were multiplied on sterilized sorghum grains to get enough inoculum. Artificial inoculation was done with a spore mixture (approximately 10^5^ spores ml^–1^) of virulent SB isolates grown on sorghum grains at booting stage (GS 43–49) twice during evening hours by using knapsack sprayer (Chaurasia et al., 1999; Joshi and Chand, 2002; Kumar et al., 2007). Experimental plots were flood irrigated after inoculation to create a conducive environment for infection and disease development. The SB severity was rated on each genotype using double-digit (00 to 99) method according to Nagarajan and Kumar (1998). The first and second digits indicates percent area with disease on flag leaf (F), and below flag leaf (F-1). Final SB severity was scored at GS 83–85 at both locations (Zadoks et al., 1974). During 2014–2015 crop season, the panel was screened again at BHU, Varanasi and disease severity was recorded three times at 5 days interval during March 2015 at GS 77–87 and area under the disease progress curve (AUDPC) at BHU was calculated (Jeger and Viljanen-Rollinson, 2001).

(1) $$A\,U\,D\,P\,C=\sum_{i=1}^{n}\left[\left(S\,B_{i+1}+S\,B_{i}\right)/2\right]\,\left[\left(t_{i+1}-t_{i}\right)\right]$$

Where, *SB*_i_ is the spot blotch severity on *i*^th^ days, *t*_i_ is the time in days at *i*^th^ observation, and *n* is the total number of observations.

The genotypes were categorized into different groups based on length of spots and hallowing (reaction type), extent of disease severity level (Double digit, i.e., on Flag and Flag-1) based on the maximum score on genotype as well as based on AUDPC values (Supplementary Table S2).

### Genotyping, Population Structure, and Linkage Disequilibrium

The 261 genotypes of the HI-AM panel were genotyped with DArT-Seq technology (Diversity Array Technology Pty Ltd., DArT P/L). The final marker sets (13182 PAVs and 6311 SNPs, respectively) were obtained by removing heterozygous and monomorphic markers and markers with minor allele frequencies (MAF) < 5% and markers with missing data > 10%. Markers distribution across the seven barley chromosome is shown in Supplementary Figure S1. Population structure was determined by using STRUCTURE version 2.3.4 (Pritchard et al., 2000), the number of subgroups was confirmed using Bayesian Information Criteria (BIC), generated with the *adegenet* package for R statistical software (The R Development Core Team). Finally, based on principal component analysis (PCA), genotypes were assigned to subgroups or considered admixed on the basis of 80% membership criterion. Linkage disequilibrium (LD) was calculated with TASSEL 5.2.32 (Bradbury et al., 2007). The extent of LD was estimated by non-linear regression analysis on the basis of intra chromosomal *r*^2^ values (Hill and Weir, 1988; Remington et al., 2001) using *nlstools* package for R Statistical Software (The R Development Core Team). More information regarding genotyping population structure and LD analysis was reported by Visioni et al. (2018).

### Genome Wide Association Mapping

Genome wide association mapping was performed combining genotypic data and disease severity scores at the seedling and adult plant stages. Genome scans were performed using both General Linear Model (GLM) and Mixed Linear Model (MLM), the general equations for GLM and MLM were reported by Visioni et al. (2018). Genomic scans using the GLM model were performed incorporating population structure (GLM + PCA model) or the Q-matrix (GLM + Q model) as covariate in order to avoid type I errors. The MLM model consider the familiar relatedness (the K model) and it was used to take into account both population structure and familiar relatedness (Q + K and PCA + K models). The kinship matrix (K) was estimated using Tassel V 5.2.32 from the both whole sets of markers. For both GLM and MLM analysis a threshold of (–log_10_ *p* ≥ 3) was set for identifying significant marker-trait associations. Significant markers mapping within the interval of LD decay were considered as being linked to the same QTL and the marker with the highest *p-value* was chosen as representing the QTL. Considering the stringency of the model used for accounting population structure, in which most of the false positives were inherently controlled. The critical *p*-value for marker-trait association was firstly determined according to a liberal approach proposed by Chan et al. (2010) rather than using false discovery rate. Considering this approach, markers were declared significant at the *p* = 0.0001 [−log(*p*) = 4] with the selected models (Visioni et al., 2018). A further step to increase confidence in QTL identified was done by applying the LD adjusted Bonferroni, proposed by Duggal et al., 2008. The value calculated for LD decay of 4 cM (Visioni et al., 2018), corresponding to 4.3 Mbp, indicated that this association panel interrogated the 987.65 cM of our association mapping panel via 246 “loci hypothesis,” and hence the Bonferroni correction for this panel was set to 3.68 −log(*p*) (*p* < 0.05).

### QTL Alignment and Candidate Genes

QTL detected for SB resistance were aligned with those previously reported in different barley germplasm by checking the position of markers at the QTL peak in the barley pseudomolecules Morex V.2.0 database. Markers sequences were mapped in the database using the IPK Barley Blast Server^1^. The position of the marker representative of the QTL was compared with those of markers at QTL peaks reported in previous studies and considered adjacent on the base of LD value (intervals selected correspond to 4 cM on each side of the QTL peak). Molecular markers sequences were aligned to the barley physical genome^2^. Putative candidate genes were then identified searching within the genes aligned and located within the LD interval at both sites of the markers at QTL peaks using PGSB database (Plant Genome System Biology^3^). The database provides access to the barley gene annotation described by the IBSC (2012). Candidate genes (CG) search was focused mainly on functional domains or genes functionally related with disease resistance mechanisms.

## Results

### Seedling Resistance to Moroccan Spot Blotch Isolates

**FIGURE 1:**
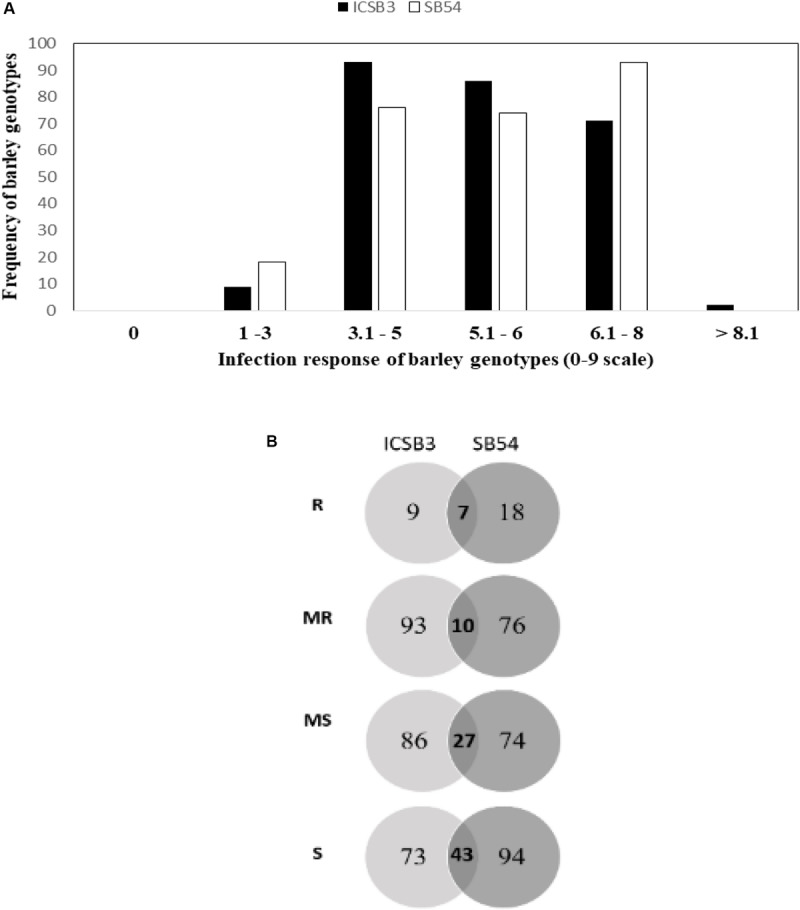
Frequency distribution of spot blotch resistance in 261 barley genotypes of HI-AM mapping panel at the seedling stage for isolates SB54 and ICSB3 **(A)**. Venn diagram of infection responses at seedling stage of 261 barley genotypes of HI-AM to two isolates of spot blotch under controlled conditions. Here, R, resistant; MR, moderately resistant; MS, moderately susceptible; S, susceptible **(B)**.

In the greenhouse, SB infection was uniform and reliable infection responses (IR) were recorded. The frequency distribution of IR HI-AM panel (261 genotypes) at the seedling stage has been presented in Figure 1. Details about the IR of individual genotype from HI-AM are available as Supplementary Figure S1. The mean IR for ND B112 (resistant check) and Annoucer (susceptible check) varied from 2.5 to 4.0 and 7 to 8.5, respectively. Of the 261 barley genotypes tested, none of them were immune to isolate SB54 (Pathotype 3) and ICSB3 (Pathotype 7). The distribution of IR of barley genotypes to isolates ICSB3 and SB54 was negatively skewed toward MR, MS, and S categories.

Interestingly, 9 genotypes (3.5%) were resistant (IR 1-3) to the isolate ICSB3, whereas, 18 genotypes (7%) were resistant to SB54. While 93 genotypes (36%) were moderately resistant (IR 4-5) to the isolate ICSB3, and 76 genotypes (29%) were moderately resistant to the isolate SB54 (Figure 1 and Supplementary Table S3).

### Adult Plant Resistance to Spot Blotch Pathogen Population in the Field

**FIGURE 2:**
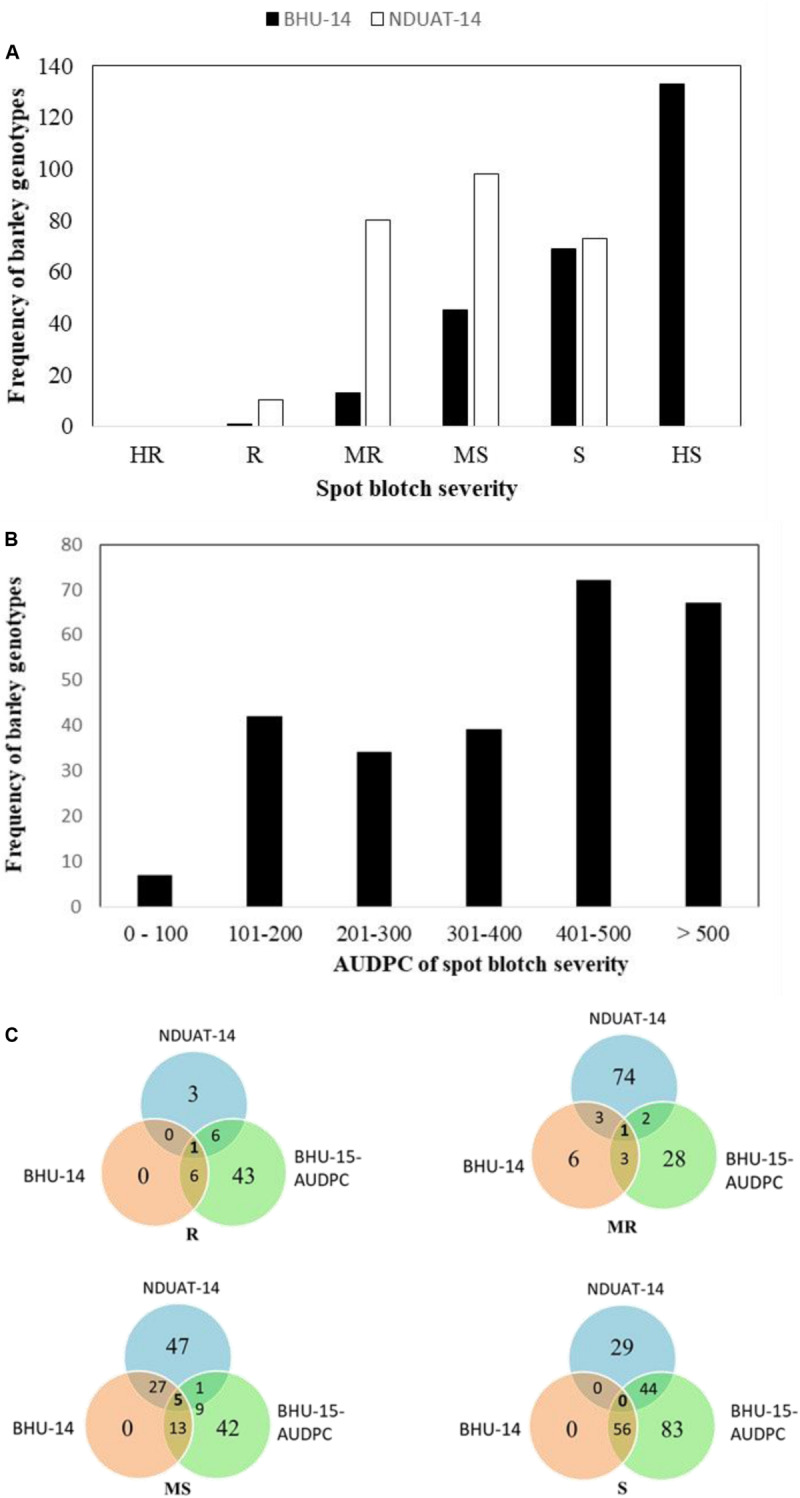
Frequency distribution of spot blotch disease severities at adult plant in Banaras Hindu University (BHU) Varanasi and Narendra Dev University of Agriculture and Technology (NDUAT) Faizabad, India during 2014 **(A)** and Area under the disease progress curve (AUDPC) of spot blotch in BHU, Varanasi, India, in 2015 **(B)**. Venn diagram representing genotypes distributions across different reaction types to spot blotch disease at the different testing sites. Here, R, resistant; MR, moderately resistant; MS, moderately susceptible; S, susceptible **(C)**.

The frequency distribution of SB severity of the HI-AM at BHU-14 (Varanasi) and NDUAT-14 (Faizabad) are presented in Figure 2A and AUDPC of SB at BHU in Figure 2B. Final SB disease severity and AUDPC of individual barley genotype is presented in Figure 2 and in Supplementary Table S4.

At BHU-14, higher disease severity of 74 ± 15 (mean ± SD) was recorded than at NDUAT-14 (55 ± 11). Furthermore, overall SB disease severity in NDUAT-14 was slightly higher in two-row barley (56.9 ± 9.9) than in six-row types (50.6 ± 10.7). But in BHU-14, the overall SB disease severity in six-row was slightly higher (75.5 ± 14.2) than two-row types (72.5 ± 15.6). Similarly, in BHU-15-AUDPC, the overall AUDPC value of 399 ± 176 was observed. The AUDPC for six-row was significantly higher (425.8 ± 175.4) than in two-row types (385.3 ± 175.6). Only three barley genotypes, HI-AM-3 (Issaria, two-row), HI-AM-241 (ZIGZIG/BLLU//PETUNIA1, six-row), and HI-AM-250 (M104/TOCTE, six-row) were found resistant and/or moderately resistant across three locations in two cropping seasons.

### Genome Wide Association Mapping

Performing GWAM for SB at SRT, the GLM procedure using PCA for accounting population structure and relatedness was the best fitting model, when analyzing data for isolate SB54 using both PAVs and SNPs markers sets. On the other hand, analyzing data for isolate ICSB3 GLM + PCA was again the best fitting model using the SNPs marker set, while the MLM procedure using PCA + K model was the best fitting model using the PAVs marker set. The genome scans for isolate SB54 showed 9 QTL located on chromosomes 1H, 3H, 4H, 6H and 7H (Table 1). Markers *R*^2^ for isolate SB54 ranged from 4.53% to 6.82% and the total phenotypic variance explained by 9 QTL was 49.26%. The GWAM analyses at SRT for ICSB3 identified 14 QTL located on chromosomes 1H, 3H, 4H, 6H and 7H (Table 1) with *R*^2^ ranging from 4.32% to 7.79%, explaining together 72% of phenotypic variance.

**TABLE 1:** GWAM for seedling resistance against two *C. sativus* isolates in Morocco based on infection response (0–9 scale).

**QTL id**	**Isolate**	**Marker**	**Chr**	**cM**	**−Log_10_(*p*)**	**Marker *R*^2^**	**Marker eff.**	**MAF**
*SRT-ICSB3-1*	ICSB3	DArT529	1	45	3.16	4.72%	0.73	21.09%
*SRT-ICSB3-2*	ICSB3	SNP520	1	120	3.23	4.59%	−1.01	9.84%
SRT-ICSB3-3*	ICSB3	DArT4210	3	2	3.65	5.74%	−0.77	35.10%
*SRT-ICSB3-4*	ICSB3	SNP1838	3	67	3.08	4.46%	−0.66	35.89%
SRT-ICSB3-5*	**ICSB3**	**DArT5749**	**3**	**133**	**4.51**	**7.79%**	−**0.89**	**23.11%**
*SRT-ICSB3-6*	ICSB3	DArT6694	4	60	3.00	4.32%	1.15	5.81%
SRT-ICSB3-7*	ICSB3	DArT9634	6	17	3.62	5.86%	0.80	21.28%
*SRT-ICSB3-8*	ICSB3	DArT9658	6	25	3.35	5.04%	−0.65	28.69%
*SRT-ICSB3-9*	ICSB3	SNP3989	6	93	3.34	4.86%	−1.15	8.00%
*SRT-ICSB3-10*	ICSB3	DArT11126	7	3	3.28	4.94%	−0.62	34.84%
*SRT-ICSB3-11*^†^	ICSB3	DArT11173	7	10	3.11	4.78%	−0.64	32.51%
SRT-ICSB3-12*	ICSB3	SNP4686	7	116	3.63	5.25%	−0.90	18.50%
***SRT-ICSB3-13***	**ICSB3**	**SNP4986**	**unk**	**unk**	**3.68**	**5.74%**	−**1.25**	**6.78%**
*SRT-ICSB3-14*	ICSB3	SNP5983	unk	unk	3.49	5.14%	−0.70	33.73%
*SRT-SB54-1*	SB54	DArT266	1	18	3.35	5.09%	0.68	38.59%
***SRT-SB54-2***	**SB54**	**DArT475**	**1**	**38**	**3.99**	**5.85%**	−**0.92**	**15.75%**
SRT-SB54-3*	**SB54**	**DArT4187**	**3**	**2**	**4.28**	**6.82%**	−**1.08**	**15.25%**
SRT-SB54-4*	**SB54**	**DArT5749**	**3**	**133**	**3.76**	**5.89%**	−**0.86**	**23.11%**
***SRT-SB54-5***	**SB54**	**SNP2594**	**4**	**68**	**3.85**	**5.68%**	**0.97**	**13.44%**
*SRT-SB54-6*	SB54	SNP2750	4	104	3.39	5.19%	0.89	27.08%
SRT-SB54-7*	SB54	DArT9634	6	17	3.27	5.06%	0.81	21.28%
SRT-SB54-8*	SB54	SNP4686	7	116	3.55	5.16%	−0.99	18.50%
*SRT-SB54-9*	SB54	SNP6285	unk	unk	3.16	4.53%	−0.72	48.22%

*Common significant QTL between the two isolates. ^†^Common QTL between SRT and APR. QTL highlighted in bold passed the LD adjusted Bonferroni test correction.

Performing GWAM for SRT to SB, using both PAVs and SNPs markers sets, the best fitting model for NDUAT-14 (Faizabad) and BHU-14 (Varanasi) was the MLM procedure using PCA + K for accounting for population structure and relatedness. When the data from BHU-15-AUDPC (Varanasi) was used for GWAM the best fitting models were MLM Q + K for the PAVs and MLM PCA + K for the SNPs marker sets, respectively. GWAM for APS showed a total of 15 QTL using disease severity data from two locations NDUAT-14 and BHU-14 and 5 QTL in BHU-15-AUDPC (Varanasi) by using AUDPC values. At BHU-14 (Varanasi) 9 QTL were located on chromosomes 2H, 3H, 4H, 5H, and 7H with marker *R*^2^ between 4.44% and 5.84% and explaining 44.09% of the total phenotypic variance (Table 2). At NDUAT-14, six QTL were found on chromosomes 1H, 2H, 4H, and 6H in BHU-14 with marker *R*^2^ ranged from 4.64% and 9.85% explaining 38.32% of the total phenotypic variance. Furthermore at BHU-15-AUDPC, five QTL were detected on chromosome 4H, 5H, and 7H with marker *R*^2^ ranged from 4.53% and 5.69% explaining 26.42% of the total phenotypic variance (Table 2). QQ plots are shown in supplementary materials (Supplementary Figures S2–S4). Overlapping QTL at SRT were found between both isolates. The QTL were located on chromosomes 3H (2 cM and 133 cM, respectively), 6H (17 cM) and 7H (116 cM). Furthermore, QTL *SRT_ICSB3_11* for SRT located on chromosome 7H (10 cM) overlaps with a QTL for APS located on the same chromosome at 12.75 cM (*APS_Var_9*).

**TABLE 2:** GWAM for adult plant resistance to a mixture of *C. sativus* isolates in India based on disease severity (double–digit score and AUDPC).

**QTL id**	**Env.**	**Marker**	**Chr**	**cM**	**−Log_10_(*p*)**	**Marker *R*^2^**	**Marker eff.**	**MAF**
***APS_Fai_1***	**NDUAT-14**	**SNP340**	**1**	**87.00**	**6.0591**	**9.85%**	**0.14**	**13.62%**
*APS_Fai_2*	NDUAT-14	DArT3556	2	127.20	3.0395	4.62%	−0.01	28.81%
***APS_Fai_3***	**NDUAT-14**	**DArT3981**	**2**	**146.72**	**4.6478**	**6.95%**	−**0.06**	**15.42%**
*APS_Fai_4*	NDUAT-14	DArT6240	4	1.27	3.6323	5.37%	−0.07	35.20%
***APS_Fai_5***	**NDUAT-14**	**SNP3639**	**6**	**30.00**	**4.3552**	**6.90%**	**0.06**	**6.33%**
*APS_Fai_6*	NDUAT-14	DArT10608	6	100.42	3.2975	4.64%	−0.07	5.79%
*APS_Var14_1*	BHU-14	DArT2274	2	40.08	3.2865	4.60%	0.07	38.65%
*APS_Var14_2*	BHU-14	DArT3041	2	94.72	3.2255	4.44%	0.28	16.93%
***APS_Var14_3***	**BHU-14**	**DArT5301**	**3**	**83.07**	**3.8817**	**5.68%**	**0.14**	**42.62%**
*APS_Var14_4*	BHU-14	SNP2134	3	128.00	3.5298	4.96%	0.12	26.98%
*APS_Var14_5*	BHU-14	DArT6861	4	79.76	3.1495	4.42%	0.09	20.00%
*APS_Var14_6*	BHU-14	DArT7465	5	35.10	3.1397	4.52%	−0.09	36.78%
*APS_Var14_7*	BHU-14	DArT7503	5	41.56	3.4649	4.87%	−0.03	34.13%
*APS_Var14_8*	BHU-14	DArT8678	5	137.22	3.4497	4.76%	−0.14	44.92%
***APS_Var14_9***^†^	**BHU-14**	**DArT11239**	**7**	**12.75**	**4.0345**	**5.84%**	**0.11**	**38.06%**
***APS-AUDPC-1***	**BHU-15**	**SNP2309**	**4**	**20.00**	**3.7618**	**5.69%**	−**100.81**	**51.39%**
*APS-AUDPC-2*	BHU-15	DArT7177	4	112.00	3.3894	5.30%	−104.76	37.50%
***APS-AUDPC-3***	**BHU-15**	**DArT8036**	**5**	**82.00**	**3.6931**	**5.52%**	−**133.57**	**13.79%**
*APS-AUDPC-4*	BHU-15	DArT12922	7	127.00	3.0703	4.53%	108.10	27.31%
*APS-AUDPC-5*	BHU-15	SNP4893	unk	unk	3.6074	5.38%	−189.60	5.47%

*^†^Common QTL between SRT and APR. QTL highlighted in bold passed the LD adjusted Bonferroni test correction.*

### Known Co-segregating Loci and Candidate Genes for Resistance to Spot Blotch

Out of the 15 QTL for SB resistance at SRT for isolate ICSB3, four were coincident with prior reports and those QTL were identified at both SRT and APS stages using different germplasm and different isolates (Zhou and Steffenson, 2013; Afanasenko et al., 2015; Gutierrez et al., 2015; Bykova et al., 2017; Wang et al., 2017). Furthermore, the QTL *SRT-ICSB-12* overlaps with a QTL detected in the same panel for stripe rust and reported in Visioni et al. (2018). Within the 9 QTL identified for isolate SB54, *SRT-SB54-6 was* already reported in a previous studies (chromosome 4H 104 cM) using different isolates and genotypes with different genetic background (Tamang et al., 2015), while QTL *SRT-SB54-65* and *SRT-SB54-8* were already reported for stripe rust in the same panel (Visioni et al., 2018). For APS resistance we found a total of 15 QTL (6 at NDUAT-14, 9 at BHU-14) and 5 QTL for AUDPC based resistance at BHU-15. Out of the 6 QTL detected at NDUAT-14, *APS-Fai-1* (chromosome 1H cM 87) was already reported at both SRT using different germplasm and different isolates (Tamang et al., 2015). QTL *APS-Var14-3* and *APS-Var14-4* (chromosome 3H cM 87 and 128, respectively) detected in BHU-Varanasi in 2014 were already reported by Tamang et al. (2015) and by Afanasenko et al. (2015) for resistance at SRT. *APS-AUDPC-1* (4H 20 cM), *APS-AUDPC-3* (5H 82 cM) and *APS-AUDPC-4* (7H 127 cM) were earlier reported by Gutierrez et al. (2015) at APS and by Tamang et al. (2015) and by Afanasenko et al. (2015), respectively.

**FIGURE 3:**
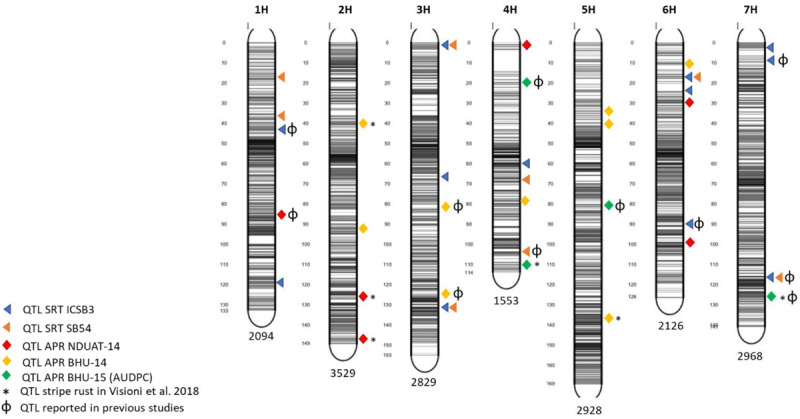
Marker distribution across the seven barley chromosomes and QTL position in the barley genome. The number of markers for each chromosome is indicated at the bottom while gray bars shows the marker density at each chromosome. The figure was produced using the Sommer package (https://CRAN.R-project.orh/package=sommer).

Five QTL detected for APS resistance were found also to overlap with others already reported for stripe rust by Dracatos et al. (2016) and by Visioni et al. (2018): *APS-Fai-3* detected at NDUAT-14, *APS-Var14-1, APS-Var14-2 and APS-Var14-8* detected at BHU-14 and *APS-AUDPC-2 at* BHU-AUDPC-15 (Table 3). An overview of QTL mapped at both SRT and APR stage is given in Figure 3

**TABLE 3:** QTL aligned and candidate genes identified for seedling and adult plant stages.

**QTL id**	**Chr**	**cM**	**Gene identifier**	**Description**	**Know co-segregating loci based**
***a) Seedling stage***					
***Isolate ICSB3***					
*SRT-ICSB3-1*	1	45	MLOC_14910.1	–	11_10275 Zhou and Steffenson (2013) (AP); 11_10275 Gutierrez et al. (2015) (AP); SCRI_RS_189483 Wang et al. (2017) (SRT), 11_10764 Afanasenko et al. (2015) (SRT)
SRT-ICSB3-3*	3	2	AK365963	NBS-LRR disease resistance protein homolog	–
SRT-ICSB3-5*	**3**	**133**	**MLOC_64418.1**	**NBS-LRR disease resistance protein family-3**	**–**
*SRT-ICSB3-6*	4	60	AK356118	Glucan endo-1,3-beta-glucosidase 4	–
SRT-ICSB3-7*	6	17	MLOC_76542.1	NB-ARC domain-containing disease resistance protein	–
*SRT-ICSB3-8*	6	25	AK371644	Glucan endo-1,3-beta-glucosidase 4	–
*SRT-ICSB3-9*	6	93	MLOC_58499.1	MYB transcription factor	11_10015 Afanasenko et al. (2015) (SRT)
*SRT-ICSB3-10*	7	3	MLOC_38445.1	NBS-LRR disease resistance protein homolog	
SRT-ICSB3-11^†^	7	10	MLOC_22072.1	NBS-LRR disease resistance protein-like protein	JHI-Hv50k-2016-445854 Bykova et al. (2017) (SRT)
SRT-ICSB3-12*	7	116	MLOC_3420.1	MYB transcription factor	11_21229 Afanasenko et al. (2015) (SRT); *SNP4686 (Id:3259386), stripe rust*, Visioni et al. (2018)
***Isolate SB54***					
*SRT-SB54-1*	1	18	MLOC_70910.1	NB-ARC domain-containing disease resistance protein	–
***SRT-SB54-2***	**1**	**38**	**MLOC_11791.2**	**Disease resistance protein**	
SRT-SB54-3*	**3**	**2**	**AK365963**	**NBS-LRR disease resistance protein homolog**	**–**
SRT-SB54-4*	**3**	**133**	**MLOC_64418.1**	**NBS-LRR disease resistance protein family-3**	**–**
***SRT-SB54-5***	**4**	**68**	**MLOC_13295.1**	**–**	***SNP2594 (Id:4174417), stripe rust*, Visioni et al. (2018)**
*SRT-SB54-6*	4	104	-	–	SCRI_RS_192689 Tamang et al. (2015) (SRT)
SRT-SB54-7*	6	17	MLOC_76542.1	NB-ARC domain-containing disease resistance protein	–
SRT-SB54-8*	7	116	MLOC_3420.1	MYB transcription factor	*SNP4686 (Id:3259386), stripe rust*, Visioni et al. (2018)
***b) Adult plant***					
*NDUAT-14*					
***APS_Fai_1***	**1**	**87.00**	**MLOC_70659.2**	**MYB TF**	**11_20792** Tamang et al. (2015) **(SRT)**
***APS_Fai_3***	**2**	**146.72**	**MLOC_6943.1**	**–**	***Dart3981 (Id: 5240790), stripe rust***, Visioni et al. (2018)
*APS_Fai_4*	4	1.27	MLOC_10090.4	NBS-LRR disease resistance protein homolog	–
***APS_Fai_5***	**6**	**30.00**	**AK371644**	**Glucan endo-1,3-beta-glucosidase 4**	**–**
*APS_Fai_6*	6	100.42	MLOC_66753	MYB family transcription factor	–
***BHU-14***					
*APS_Var14_1*	2	40.08	MLOC_73232.1	CsAtPR5 pathogenesis response	*DaRT2274 (Id: 4790278), stripe rust*, Visioni et al. (2018)
*APS_Var14_2*	2	94.72	MLOC_74624.1	–	*DaRT3041 (Id: 4785201), stripe rust*, Visioni et al. (2018); Dracatos et al. (2016) *(stripe rust)*
***APS_Var14_3***	**3**	**83.07**	**MLOC_34610.2**	**–**	**SCRI_RS_159340** Tamang et al. (2015) **(SRT)**
*APS_Var14_4*	3	128.00	AK369539	NBS-LRR disease resistance protein homolog	11_20920 Afanasenko et al. (2015) (SRT)
*APS_Var14_8*	5	137.22	MLOC_63574.2	Glucan endo-1,3-beta-glucosidase 5	*DaRT8678 (Id: 3267960), stripe rust*, Visioni et al. (2018)
***APS_Var14_9***^†^	**7**	**12.75**	**MLOC_22072.1**	**NBS-LRR disease resistance like protein**	**–**
***AUDPC***					
***APS-AUDPC-1***	**4**	**20.00**	**MLOC_71409.1**	**–**	**11_11136 Gutierrez et al. (2015) (AP)**
*APS-AUDPC-2*	4	112.00	AK365216	Disease resistance-responsive (dirigent-like protein) family protein	*DaRT7177 (Id: 3666382), stripe rust*, Visioni et al. (2018)
***APS-AUDPC-3***	**5**	**82.00**	**MLOC_7890.1**	**Beta-glucosidase**	**11_20850 Afanasenko et al. (2015) (SRT)**
*APS-AUDPC-4*	7	127.00	MLOC_75995.1	NBS-LRR resistance-like protein	11_20847 Tamang et al. (2015) (SRT)

*Common significant QTL between the two isolates. ^†^Common QTL between SRT and APR. QTL highlighted in bold passed the LD adjusted Bonferroni test correction. Know co-segregating loci indicated in italic refers to previous QTL mapped in the same position for stripe rust (Visioni et al., 2018).

Candidate genes identified for QTL at both SRT and APR are reported in Table 3. Most of the QTL detected were located in regions enriched with functional domains or genes involved in host plant defense based upon their annotation. In total, we have identified 26 CG (15 at SRT and 11 at APS, respectively) for SB resistance, most of the CG shows homology with resistance genes belonging to nucleotide binding sites with leucine rich repeat (NBS-LRR) class, disease resistance proteins, MYB transcription factors and genes involved in β-glucans biosynthesis.

## Discussion

### Host Resistance to SB

In this study, we have used two Moroccan SB isolates; pathotype 3 (SB54) and pathotype 7 (ICSB3) to map loci conferring seedling resistance in a diverse barley germplasm from ICARDA, adapted specifically to the high input conditions. SB was not reported in Morocco until recently our group found this disease during disease survey in 2015 (unpublished data) Net Form of Net Blotch (NFNB) and Spot Form of Net Blotch (SFNB) have been prevalent with disease incidence up to 70% and disease severity from 40 to 90%, respectively (Yousfi and Ezzahiri, 2002; Jebbouj and Brahim, 2010; Gyawali et al., 2018; Rehman et al., unpublished data). The pathotype information of Moroccan SB isolates was unknown until our studies revealed that highly virulent pathotypes 7 was found along with other pathotypes 0, 1, and 2. Previous mapping studies of SB resistance in barley have used pathotype 1, 2, or 7 (Bilgic et al., 2006; Roy et al., 2010; Wang et al., 2017). To our knowledge this is the first study where pathotype 3 has been used for screening barley HI-AM to identify SRT QTL. The identification of pathotype 7 isolate from Morocco is quite alarming for stable barley production because six-row barley landraces are widely grown under low input conditions by many small holder farmers of Morocco. Our results suggest that most of the barley genotypes grown in Morocco are very susceptible to SB54 and ICSB3 (Rehman et al., unpublished data).

In SRT, 18 (7%) genotypes showed resistance reaction to isolate SB54 and 7 genotypes (2.7%) were resistant to isolate ICSB3 (Figure 1 and Supplementary Table S3). However, previous SRT study of barley association mapping panel (AM-2014) at ICARDA, revealed only 1 out of 336 barley genotypes to be resistant to a mixture of 19 *C. sativus* isolates from Morocco (Gyawali et al., 2018). This can be explained due to the presence of diverse repertoire of avirulence genes from all SB isolates on a diverse barley germplasm which can mask the detection of gene-for-gene interactions. About 78% (14 out of 18) resistant barley genotypes in case of SB54 and 86% (6 out of 7) genotypes in case of ICSB3 were of two-row type. Interestingly, seven barley genotypes (HI-AM-85, 88, 89, 95, 199, 216, 218) were resistant to both pathotypes with six out of seven lines being two-row type. The over representation of two-row type resistance to both SB pathotypes might be the first report of its kind. This can be also explained by the absence of population subgrouping based on ear type and/or by the fact that ICARDA’s breeding program routinely exercise hybridization between two-row and six-row types (Visioni et al., 2018).

Spot blotch is also a major constraint for barley production in South Asian countries like China, Nepal, India, Bangladesh, and Pakistan due to hot and humid climate prevailing during February to March (Dubin and van Ginkel, 1991; Kumar et al., 2007; Chand et al., 2008; Singh et al., 2009; Vaish et al., 2011; Prasad et al., 2013). More specifically, in the North Eastern Indian states (Uttar Pradesh, Bihar, and Jharkhand), the winter is very short and relatively warmer weather provides perfect conditions for SB. Singh et al. (2009) reported yield loss of 79.6% in susceptible cultivar RD2503 in India (Singh et al., 2009). BHU (Varanasi) has been used as SB hot spot for screening wheat and barley germplasm (Chand et al., 2008; Prasad et al., 2013; Gyawali et al., 2018). In our study, we have also found that the disease severity was much higher at BHU-Varanasi (74 ± 15 in 2014 and 64 ± 23 in 2015) than in NDUAT-Faizabad (55 ± 11 in 2014) which corroborate findings of Gyawali et al. (2018). This can be attributed to high inoculum pressure, disease conducive environment, and existence of more virulent SB races at BHU-Varanasi than at NDUAT-Faizabad. Unfortunately, SB pathotypes in India are poorly characterized and SRT studies with pure isolates are lacking. We found that rating genotypes by double-digit scale based on final observation (highest reaction) seems to indicate fewer SB resistant barley genotypes in field screening as compared to AUDPC where relative disease progress is recorded at three timepoints. For example, at NDUAT-14 and BHU-14, only 1 and 10 (4%) genotypes, respectively, were found resistant based on single observation on the highest disease score as compared to BHU-15-AUDPC, where 49 (19%) genotypes were found resistant to SB (Figure 2 and Supplementary Table S4). Gyawali et al. (2018) reported 6.5% (22 genotypes) to be resistant at BHU, in the AM-2014, while in HI-AM we observed 19% (49 genotypes) resistant genotypes at BHU with AUDPC observations. Thus HI-AM offers much more diversity for SB resistance breeding program of India.

### Genome Wide Association Mapping for SB Resistance

For pathotype 7 isolate (ICSB3), chromosome 1H harbors two SRT QTL on 45 cM (*SRT-ICSB3-1*) and 120 cM (*SRT-ICSB3-2)*. *SRT-ICSB3-1* was already reported in SRT by Afanasenko et al. (2015) and by Wang et al. (2017) and at APR in the USDA barley core collection, and in another association mapping panel, respectively (Zhou and Steffenson, 2013; Gutierrez et al., 2015). Furthermore, on chromosome 3H, three SRT QTL *SRT-ICSB3-3* (2 cM), *SRT-ICSB3-4* (67 cM), and *SRT-ICSB3-5* (133 cM) explained a total of 18% phenotypic variation. All identified QTL remained to be novel.

On chromosome 6H, three SRT QTL *SRT-ICSB3-7* (17 cM), *SRT-ICSB3-8* (25 cM), and *SRT-ICSB3-9* (93 cM) explained an overall phenotypic variation of 15.76%. The QTL *SRT-ICSB3-9* (93 cM) was already reported in seedling resistance to diverse SB isolates by Afanasenko et al. (2015). Similarly, on chromosome 7H, three SRT QTL *SRT-ICSB3-10* (3 cM), *SRT-ICSB3-11* (10 cM) and *SRT-ICSB3-12* (116 cM) explained 15% phenotypic variation against ICSB3 isolate. Further, two SRT QTL *SRT-ICSB3-11* (10 cM) and *SRT-ICSB3-12* (116 cM) have been reported to be involved in seedling resistance to different SB isolates and in diverse barley germplasm in two different studies by Afanasenko et al. (2015) and Bykova et al. (2017). *SRT-ICSB3-12* also overlaps with a QTL for stripe rust resistance reported by Visioni et al. (2018).

For pathotype 3 isolate (SB54), two novel QTL located on chromosome 3H two *SRT-SB54-3* (2 cM), and *SRT-SB54-4* (133 cM) explained together the 12.75% of phenotypic variation. Furthermore on chromosome 4H, *SRT-SB54-5* (68 cM), overlaps with a previous QTL mapped for stripe rust resistance by Visioni et al., 201, while *SRT-SB54-6* (104 cM) had also shown to be involved in SRT (Tamang et al., 2015). The single QTL *SRT-SB54-7* (17 cM) on chromosome 6H and on chromosome 7H *SRT-SB54-8* (116 cM), to the best of our knowledge have never been reported. *SRT-SB54-8* also overlaps with a previous QTL mapped by Visioni et al. (2018) for stripe rust.

Common SRT QTL to pathotype 3 (SB54) and pathotype 7 (ICSB3) were identified in this study. Two SRT QTL on 3H (2 and 133 cM), one on 6H (17 cM) and one on 7H (116 cM) explained a total phenotypic variation of 24.64% to ICSB3 and 22.83% to SB54, respectively. Strikingly, except SRT QTL on chromosome 6H (*SRT-ICSB3-7, SRT-SB54-7*), all three SRT QTL on chromosome 3 and 7H showed negative effect, thus conditioning resistance response to both SB isolates tested.

In case of adult plant stage resistance, one QTL on 1H (*APS-Fai-1*) at 87 cM in NDUAT-14 showed the highest R^2^ explaining 9.85% of the variance. *APS-Fai-1* was already reported to be involved in seedling resistance (Tamang et al., 2015). In addition, four novel QTL were found significantly associated with APR on chromosome 2H. A QTL from NDUAT-14, *APS-Fai-3* (146.72 cM), and two detected at BHU-14, *APS-Vars-14-1* (40.08 cM) QTL *APS-Vars-14-2* (94.72 cM) were already reported for stripe rust resistance in the same association mapping panel by Visioni et al. (2018).

No QTL were found at NDUAT-14 on chromosome 3H while at BHU-14, two APR QTL *APS-Var-14-3* (83.07 cM) and *APS-Var-14-4* (128 cM) were found and both have been reported to be involved in SRT to SB (Afanasenko et al., 2015; Tamang et al., 2015). Chromosome 4 harbors four APS QTL, *APS-Fai-4* (1.27 cM) at NDUAT-14, *APS-Var-14-5* (79.76 cM) at BHU-14, and *APS-AUDPC-1* (20 cM) and *APS-AUDPC-2* (112 cM) at BHU-AUDPC-15. Only one QTL *APS-AUDPC-1* (20 cM) has been reported to be involved in APR by Gutierrez et al. (2015) while other three QTL are novel. Furthermore, 3 novel APS QTL were mapped on chromosome 5H at BHU-14, *APS-Var-14-6* (35.1 cM), *APS-Var-14-7* (41.56 cM) and *APS-Var-14-8* (137.22 cM). Moreover, BHU-AUDPC-15 contributed with one more QTL on 5H, *APS-AUDPC-3* (82 cM) that was already reported by Afanasenko et al. (2015). Furthermore *APS-Var-14-8* was already associated to stripe rust resistance and showed to be stable across years and environments (Visioni et al., 2018). At NDUAT-14, on chromosome 6H two novel APR QTL *APS-Fai-5* (30 cM), and *APS-Fai-6* (100.42 cM) were detected. Whereas, on chromosome 7H two QTL were found; one at BHU-14, *APS-Var-14-9* (12.75 cM), the second QTL was detected at BHU-15 (*APS-AUDPC-415-4* at 127 cM), the first remained to be novel while the second was already reported by Tamang et al. (2015)

Only one common QTL could be detected on chromosome 3H to ICSB3 (133 cM) and SB54 (133 cM) at SRT, and at APR in BHU-14 (128 cM). Furthermore, both SRT (*SRT-ICSB3-11)* and APR QTL (*APS-Var-14-9*) are located on chromosome 7H at 10 and 12.75 cM, respectively, and hence could represent the same QTL.

### Candidate Genes

In case of SRT, 15 QTL out of 23, and in case of APR, 11 QTL out of 20 showed association with functional candidate genes. The genomic regions where most of the QTL have been mapped seems to be enriched with NBS-LRR disease resistance like proteins (10/15 SRT CG; 4/11 APS CG), pathogenesis related proteins (2/15 SRT CG; 4/11 APS CG), and MYB transcription factors (3/15 SRT CG; 1/11 APS CG). NB-LRR disease resistance proteins have been implicated in effector triggered immunity to various pathogens and a similar role to SB resistance is envisaged here. Nucleotide-binding (NB)-LRR (leucine rich repeat) proteins (NLRs) have been associated with quantitative resistance to necrotrophs. A combination of transcriptomics and association mapping of pathogen or hosts will result in the identification of novel necrotrophic effectors (NEs) and corresponding QTL, respectively. A probable strategy would be to eliminate host plant susceptibility genes for both biotrophic and necrotrophic pathogens. Furthermore, minor R genes (APR) could be pyramided for durable control of diverse pathogens (Virdi et al., 2016; See et al., 2018).

The CG associated with the peak marker DArT6694 QTL (*SRT_ICSB3_6*) on 60 cM (1H) and DArT9658 marker (*APS_Var14-8*) on 137.22 cM of chromosome 5H is a glucan endo-1,3-beta-glucosidase-4 like protein and it has more than 90% amino acid identity with orthologs from rice, maize, sorghum, wheat. The glucan endo-1,3-beta-glucosidase belongs to the family of PR proteins which are induced upon pathogen ingress. These beta-1,3-glucanases targets (1,3)-beta-D-glucosidic linkages of glucans present in fungal cell wall and enhance fungal resistance in crop plants (Kasprzewska, 2003; Kirubakaran and Sakthivel, 2007). Similarly, the peak markers of three SRT QTL (*SDL_ICSB-9*, 6H, 93 cM; *SDL_ICSB-12*, 7H, 116 cM; *SDL_SB54-8*, 7H, 116 cM) and one APR QTL (*APS_Fai_1*, 87 cM, 1H) are closely associated with MYB transcription factors. Further, MYB transcription factor (MYB15) was implicated as a regulator of defense-induced lignification and basal immunity to bacterial pathogen *Pseudomonas syringae* (Chezem et al., 2017). In addition, MYB TF are key factors in regulatory networks and respond to biotic stresses (Dubos et al., 2010). Depending upon its functional annotation, it can be envisaged that MYB TF can induce defense response to SB in adult plants. Considering the high phenotypic variation of 10%, the SNP340 marker (*APS_Fai_1*, 87 cM, 1H) could be a potential candidate for MAS.

The putative candidate gene (CG) associated with the DArT2274 marker (*APS_Vars_14-1*) on 2H at 40.08 cM encodes CsAtPR5 pathogenesis- related (PR) protein. PR proteins are conserved in many plant species and are induced upon biotic stresses conditioned by various pathogens (Prasath et al., 2014). The wheat ortholog *TaAetPR5* is 93% identical to *CsAtPR5* and was upregulated upon infection of *Blumeria graminis* f.sp. *tritici* only in the resistant line (Niu et al., 2007). Likewise, enhanced expression of PR genes was observed in resistant barley upon inoculation with *P. teres teres* (Al-daoude et al., 2017).

## Conclusion

One two-row barley genotype HI-AM-32 (Issaria) was recorded as resistant and two six-row barley genotypes, HI-AM-241 (ZIGZIG/BLLU//PETUNIA 1) and HI-AM-250 (M104/TOCTE), were found as moderately resistant across two locations during two cropping seasons. The present study has further unlocked the genetic potential of HI-AM with the identification of 15 novel QTL for SRT and 14 novel QTL for APR. Furthermore, 11 previously mapped QTL were also identified (5 for SRT and 6 for APR). Markers at QTL peak will enrich the existing allelic diversity for SB resistance and once validated, could be used for MAS to pyramid multiple resistance alleles to curb losses induced by this economically important pathogen of barley. The three lines observed as resistant/moderately resistant across the three environments can be readily utilized in barley breeding program for incorporation of effective SB resistance targeted for South Asia and North Africa.

## Data Availability Statement

The datasets presented in this study can be found in online repositories. The names of the repository/repositories and accession number(s) can be found below:

https://mel.cgiar.org/reporting/report/id/5949/del_id/20282

https://hdl.handle.net/20.500.11766.1/FK2/R2AW6A or

https://dx.doi.org/20.500.11766.1/FK2/R2AW6A

https://hdl.handle.net/20.500.11766.1/FK2/QRXB0W or

https://dx.doi.org/20.500.11766.1/FK2/QRXB0W

## Author Contributions

SR, AV, and RV conceived and coordinated the study. AV performed statistical and bioinformatics analysis. SR, SSV, SS, and RPSV collected the phenotypic data. SR, AV, RV, SG, and AA-A reviewed and contributed to the manuscript.

## Conflict of Interest

The authors declare that the research was conducted in the absence of any commercial or financial relationships that could be construed as a potential conflict of interest.

## Footnotes

1 https://webblast.ipk-gatersleben.de/barley_ibsc/viroblast.php

2 ftp://ftpmips.helmholtz-muenchen.de/plants/barley/public_data/anchoring/

3 http://pgsb.helmholtz-muenchen.de/plant/index.jsp
