# Supplementary material for: Genome Wide Association Mapping of Spot Blotch Resistance at Seedling and Adult Plant Stages in Barley
Source: Front Plant Sci. 2020 May 25;11:642. doi: 10.3389/fpls.2020.00642 (PMC7326046; doi:10.3389/fpls.2020.00642)
Supplement: Supplementary file 2 [file Data_Sheet_1.docx]

**Supplementary material**

**Suppl. Table 2.** Categorization of barley genotypes into different disease responses recorded during field trials screening against spot blotch (*Cochliobolus sativus*) of barley.

| **Reaction type** | **Length of**  **Spot (mm)** | **Disease Score**  **Double Digit**  **(Flag, Flag-1) ^†^** | **Response type** | **AUDPC^‡^** |
| --- | --- | --- | --- | --- |
| Highly Resistant | <2 | 00-24 | Small brown spots | 0-100 |
| Resistant | 2-4 | 24-34 | Medium size brown spots | 101-200 |
| Moderately Resistance | 4-6 | 35-47 | Large brown spots with little hallowing | 201-300 |
| Moderately Susceptible | 6-8 | 48-58 | Large brown blotches with prominent hallowing | 301-400 |
| Susceptible | 8-10 | 59-79 | Blotches with profuse hallowing | 401-500 |
| Highly Susceptible | >10 | >79 | Blotches with profuse hallowing | >500 |

**^†^** Disease score in double digit on Flag and next below (Flag-1) leaves at early dough stage (Growth stage 83 on Zadok’s scale); **^‡^**AUDPC= Area under disease progress curve.

**Supp. Table 3.** Infection responses at seedling stage of 261 barley genotypes of HI-AM to two isolates of spot blotch under controlled conditions. Here R= Resistant, MR= Moderately resistant, MS= Moderately susceptible, S= susceptible.

| Infection response | Infection type | ICSB3 | SB54 | Overlap |
| --- | --- | --- | --- | --- |
| I | 0 | 0 | 0 | 0 |
| R | 1 -3 | 9 | 18 | 7 |
| MR | 3.1 - 5 | 93 | 76 | 10 |
| MS | 5.1 - 6 | 86 | 74 | 27 |
| S | 6.1 - 8 | 71 | 93 | 44 |
| HS | > 8.1 | 2 | 0 | 0 |

**Supp. Table 4.** Adult plant resistance of HI-AM to spot blotch at NDUAT and BHU. Here R= Resistant, MR= Moderately resistant, MS= Moderately susceptible, S= susceptible.

| **Reaction type** | **Disease Score**  **Double Digit**  **(Flag, Flag-^1^)^†^** | **Frequency at**  **BHU14** | **Frequency at NDUAT14 14** | **AUDPC^‡^** | **Frequency** |
| --- | --- | --- | --- | --- | --- |
| Highly Resistant (HR) | 00-24 | 0 | 0 | 0-100 | 7 |
| Resistant (R) | 24-34 | 1 | 10 | 101-200 | 42 |
| Moderately Resistance (MR) | 35-47 | 13 | 80 | 201-300 | 34 |
| Moderately Susceptible (MS) | 48-58 | 46 | 97 | 301-400 | 39 |
| Susceptible (S) | 59-79 | 69 | 74 | 401-500 | 72 |
| Highly Susceptible (HS) | >79 | 132 | 0 | >500 | 67 |

**^†,^** Disease score in double digit on Flag and next below (Flag-1) leaves at early dough stage (Growth stage 83 on Zadok’s scale); **^‡^**AUDPC= Area under disease progress curve.

**Supp. Figure 1.** Markers distributions across the seven barley chromosomes

**
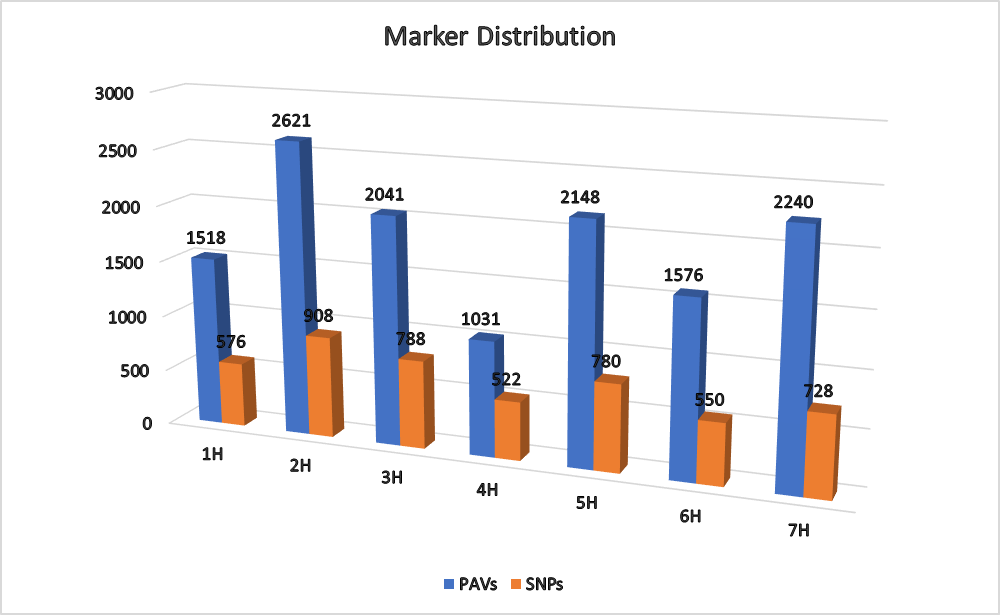
**

**Suppl. Figure S2.**

QQ plots of marker-trait association at SRT stage for (a) Isolate SB54 using PAVs markers set (GLM+PCA); (b) Isolate SB54 using SNPs markers set (GLM+PCA); (c) Isolate ICSB3 using PAVs markers set (GLM PCA); (d) Isolate ICSB3 using SNPs markers set (GLM+PCA). The black line is the expected line under the null distribution.

**
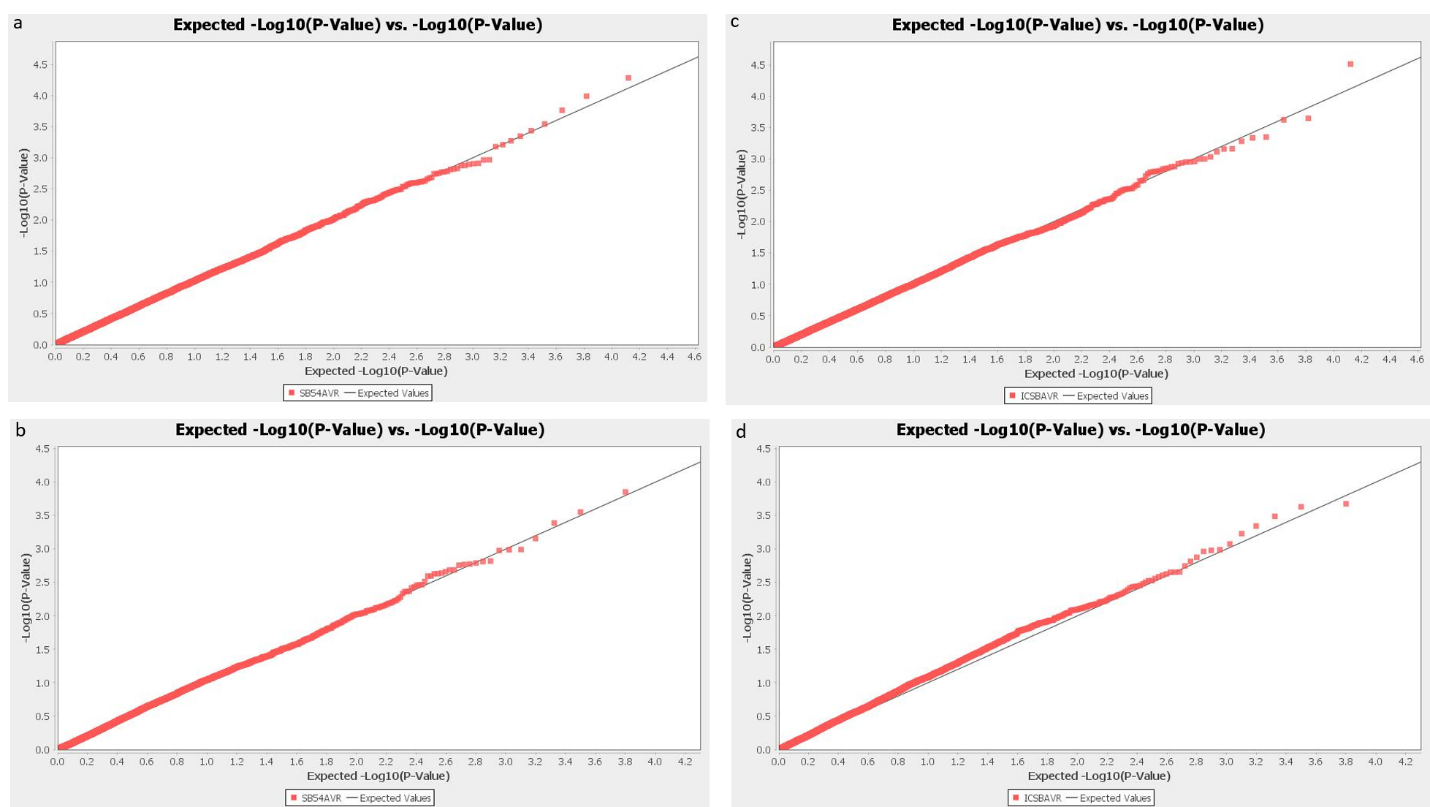
**

**Suppl. Figure S3.**

QQ plots of marker-trait association at APS using (a) PAVs markers set and the MLM PCA+K procedure for both BHU-14 and NDUAT-14 (b) SNPs markers set and MLM PCA+K procedure for both BHU-14 and NDUAT-14. The black line is the expected line under the null distribution.

**
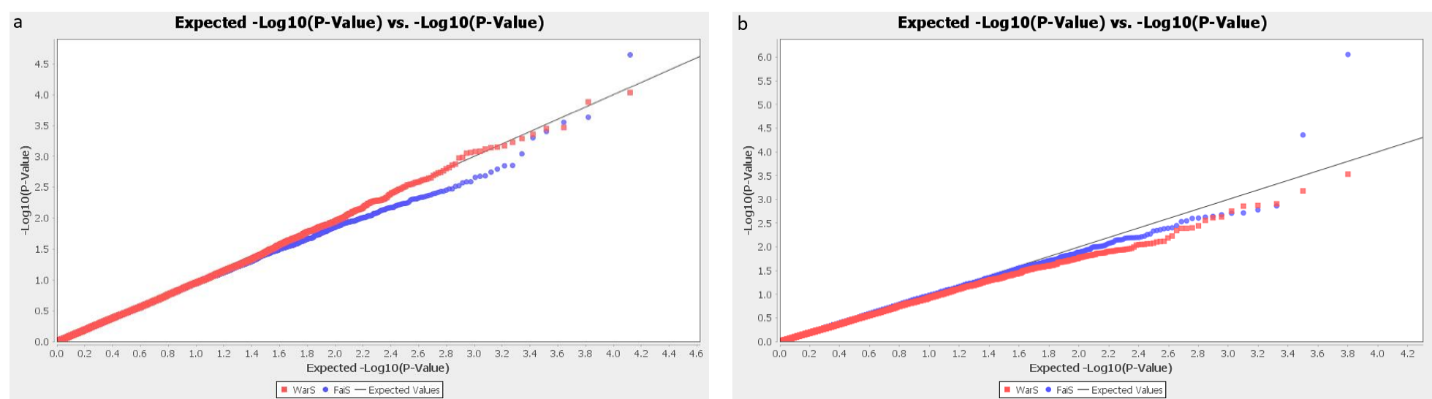
**

**Suppl. Figure S4.**

QQ plots of marker-trait association at SRT stage for: (a) PAVs markers set and the MLM (Q+K) procedure for BHU-15-AUDPC (b) SNPs markers set and MLM (Q + PCA) procedure for BHU-15-AUDPC. The black line is the expected line under the null distribution.

**
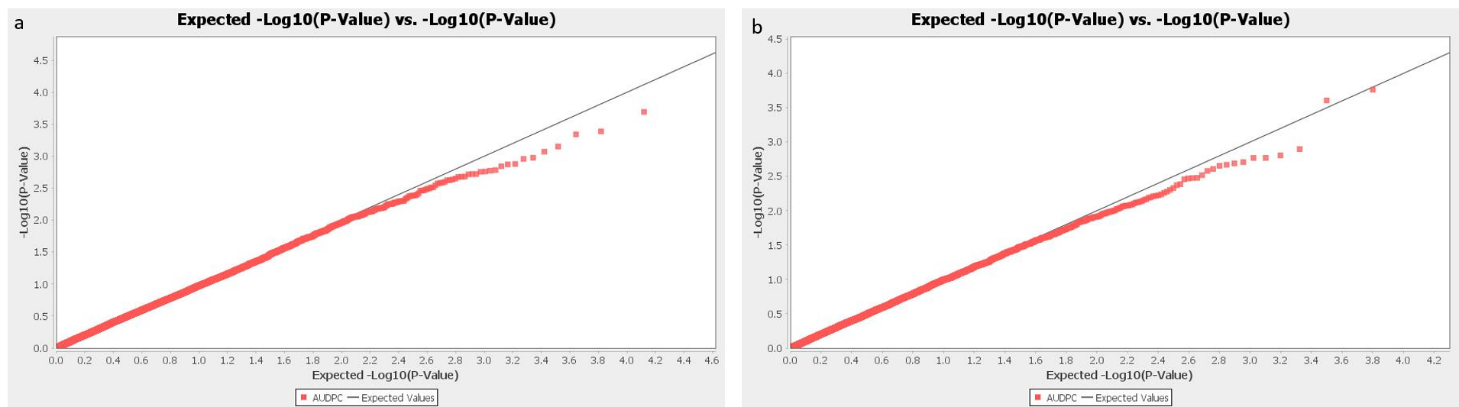
**
